# A novel single‐chain enzyme complex with chain reaction properties rapidly producing thromboxane A_2_ and exhibiting powerful anti‐bleeding functions

**DOI:** 10.1111/jcmm.14711

**Published:** 2019-10-19

**Authors:** Yan Li, Qun‐Ying Li, Qing‐Lan Ling, Shui‐Ping So, Ke‐He Ruan

**Affiliations:** ^1^ Department of Pharmacological and Pharmaceutical Sciences Center for Experimental Therapeutics and Pharmacoinformatics College of Pharmacy University of Houston Houston TX USA; ^2^ Visiting Scholar from Department of Ultrasound Second Affiliated Hospital Zhejiang University College of Medicine Hangzhou City China

**Keywords:** enzyme engineering, haemostasis, prostanoids, thromboxane A_2_

## Abstract

Uncontrollable bleeding is still a worldwide killer. In this study, we aimed to investigate a novel approach to exhibit effective haemostatic properties, which could possibly save lives in various bleeding emergencies. According to the structure‐based enzymatic design, we have engineered a novel single‐chain hybrid enzyme complex (SCHEC), COX‐1‐10aa‐TXAS. We linked the C‐terminus of cyclooxygenase‐1 (COX‐1) to the N‐terminus of the thromboxane A_2_ (TXA_2_) synthase (TXAS), through a 10‐amino acid residue linker. This recombinant COX‐1‐10aa‐TXAS can effectively pass COX‐1–derived intermediate prostaglandin (PG) H_2_ (PGH_2_) to the active site of TXAS, resulting in an effective chain reaction property to produce the haemostatic prostanoid, TXA_2_, rapidly. Advantageously, COX‐1‐10aa‐TXAS constrains the production of other pro‐bleeding prostanoids, such as prostacyclin (PGI_2_) and prostaglandin E_2_ (PGE_2_), through reducing the common substrate, PGH_2_ being passed to synthases which produce aforementioned prostanoids. Therefore, based on these multiple properties, this novel COX‐1‐10aa‐TXAS indicated a powerful anti‐bleeding ability, which could be used to treat a variety of bleeding situations and could even be useful for bleeding prone situations, including nonsteroidal anti‐inflammatory drugs (NSAIDs)‐resulted TXA_2_‐deficient and PGI_2_‐mediated bleeding disorders. This novel SCHEC has a great potential to be developed into a biological haemostatic agent to treat severe haemorrhage emergencies, which will prevent the complications of blood loss and save lives.

## 1 INTRODUCTION

Thromboxane A_2_ (TXA_2_) is one type of thromboxane, which is mainly generated by activated platelets. TXA_2_ is able to activate platelets and induce aggregation of the activated platelets.^1^ In addition, TXA_2_ has a strong ability to mediate vasoconstriction and is one of the main players in tissue injury.^2^, ^3^, ^4^, ^5^ Normally, TXA_2_ is produced through the triple‐catalytic activities: in the bleeding site, arachidonic acid (AA) released from the injured tissue is converted to the prostaglandin G_2_ (PGG_2_) and then the unstable intermediate prostaglandin H_2_ (PGH_2_) by cyclooxygenase‐1 (COX‐1); rapidly, the unstable PGH_2_ is further isomerized into anti‐bleeding TXA_2_ by the TXA_2_ synthase (TXAS) in platelets.^6^ However, the intermediate PGH_2_ could also be isomerized to prostacyclin (PGI_2_) and prostaglandin E_2_ (PGE_2_) by prostaglandin‐I and prostaglandin‐E synthases (PGIS, PGES), which have the opposite properties when compared with TXA_2_, such as antiplatelet aggregative and vasodilative properties.^6^ Thus, TXA_2_, PGI_2_ and PGE_2_ are directly involved in maintaining local haemostasis.

Generally, many bleeding emergencies can be very dangerous, and even life‐threatening. For example, arterial haemorrhage, one of the most dangerous bleeding emergencies, is always difficult to control and can result in massive blood loss in a short time. Another example is the application of aspirin, and other nonsteroidal anti‐inflammatory drugs (NSAIDs) in surgical operations or medical treatment, which strongly inhibits the COX‐1 activity, shutting down the biosynthesis of TXA_2_ in platelets, and causing dangerous bleeding situations.^7^ Aspirin, especially, can chemically modify COX‐1 and irreversibly inhibit the COX‐1 activity, which results in permanent damages to the platelet function. Fully rescuing the aspirin‐resulted TXA_2_‐deficient bleeding may take up to 7‐10 days, until the newly produced functional platelets are released from the bone marrow.^8^ Therefore, it is essential to develop a method which could be beneficial for saving lives in various bleeding emergencies.

Here, we proposed one possible effective approach to instantly handle a variety of bleeding situations and even be able to overcome aspirin‐resulted TXA_2_‐deficient bleeding disorder or PGI_2_‐mediated bleeding disorder. This novel approach was aimed to isomerize the AA (released in the bleeding site) into more TXA_2_ and simultaneously restrict the production of PGI_2_ and PGE_2_. A biological reagent with these multiple effects has not been developed yet. One of the major challenges is that the prostaglandin synthases, TXAS, PGIS and PGES, almost have equal affinities to share PGH_2_ as their common substrate.^9^ Therefore, a change in the distribution of PGH_2_ to the particular isozyme is the key to control the metabolism of AA into the specific prostanoid. In recent years, using an enzymatic engineering approach to control the distribution of PGH_2_ has been focused by our group to address this issue.^10^, ^11^, ^12^, ^13^, ^14^, ^15^, ^16^, ^17^ In our previous studies, we have successfully created a single‐chain hybrid enzyme complex (SCHEC), COX‐1‐10aa‐PGIS, through the enzymatic engineering approach, which can force AA to be isomerized into PGI_2_, in order to rescue the deficiency of PGI_2_ and to study the vascular protection effects of PGI_2_ in cellular and animal models.^10^, ^11^, ^12^, ^13^ Another SCHEC, COX‐2‐10aa‐mPGES‐1, which can effectively pass PGH_2_ to mPGES‐1, to convert AA to PGE_2_, has also been created as a model for understanding how PGE_2_ is biosynthesized during inflammation.^14^, ^15^, ^16^, ^17^, ^18^ In this study, we created a novel SCHEC, linking the C‐terminus of COX‐1 to the N‐terminus of the TXAS, through a 10‐residue amino acid linker, to directly guide the metabolism of AA to TXA_2_ by effectively passing the COX‐1 produced PGH_2_ to TXAS. COX‐1‐10aa‐TXAS demonstrated triple properties, including increasing PGH_2_ to be metabolized into the anti‐bleeding TXA_2_ and simultaneously reducing PGH_2_ to be isomerized into the pro‐bleeding PGI_2_ and PGE_2_. These triple properties could rapidly rescue the cellular deficiency of TXA_2_ and rebalance the AA metabolites in platelets to meet the needs to terminate bleeding. Therefore, this novel SCHEC has great potential to be developed into an efficient enzymatic haemostatic agent, which is able to prevent severe complications or even deaths caused by severe blood loss in various bleeding emergencies.

## 2 MATERIALS AND METHODS

### 2.1 Engineering of the SCHEC, COX‐1‐10aa‐TXAS cDNA and subcloning

The sequence of COX‐1 linked to TXAS through a 10‐aa linker (COX‐1‐10aa‐TXAS) was produced by PCR and subcloning procedures using similar methods as previously described.^19^, ^20^, ^21^, ^22^ Through the PCR cloning method, the cDNA was successfully subcloned into the pcDNA 3.1(+) vectors between the two BamHl sites containing a cytomegalovirus early promoter. The correct inserted size of cDNA was confirmed by restriction enzyme digestion analysis and DNA sequencing.

### 2.2 Cell culture and expressing the SCHEC in HEK293 cells

The human embryonic kidney cells 293 (HEK293) were purchased from ATCC. HEK293 cells were cultured in a 100‐mm cell culture dish and grown in a 37°C humidified incubator with 5% CO_2_ supply. The medium used for the culture was the high glucose Dulbecco's modified Eagle's medium containing 10% fetal bovine serum and 1% antibiotic and antimycotic.

### 2.3 Expression of the SCHEC in HEK293 cells

Stable expression of COX‐1‐10aa‐TXAS and control enzymes in HEK293 cells was performed using the previously established methods.^23^, ^24^ Briefly, the cells were cultured and transfected with a purified plasmid contained the corresponding cDNA using the Lipofectamine 2000 approach following the instructions provided by the manufacturer (Invitrogen). After 48 hours of transfection, G418 (400 μg/mL) was added to the medium for screening HEK293 cell line with stable expression ability for the recombinant proteins. The whole screening process took 4‐5 weeks.

### 2.4 Immunofluorescence staining

The immunostaining procedures were performed as previously described.^25^ Briefly, the cells cultured on cover slides were fixed and then incubated with the primary antibody (10 µg/mL COX‐1 or TXAS antibody) in the presence of 0.25% saponin. After 1‐hour incubation (room temperature), the unbound primary antibodies were washed away with PBS and then incubated with the secondary antibodies labelled with rhodamine or FITC. The stained slides were examined under the Zeiss Axioplan 2 epifluorescence microscope.

### 2.5 Examination of the enzymatic activities of SCHEC using HPLC‐scintillation analyzer method

The method was followed as previously described.^25^ Briefly, the transfected cells were washed three times and then suspended in PBS. Then, [^14^C]‐AA (3 µmol/L) was added and PBS was used to balance the total volume to 100 µL. After 5‐minute incubation, the reaction was terminated by the addition of buffer A (50 µL of 0.1% acetic acid containing 35% acetonitrile) and centrifuged at 16 500 g for 5 minutes. The C18 column (4.5 × 250 mm) was used to separate the mixture, using buffer A with a gradient of 35%‐100% of acetonitrile for 45 minutes at a 1.0 mL/min flow rate. The metabolites of [^14^C]‐AA were monitored by a liquid scintillation analyzer (Packard 150TR) built in the HPLC system.

### 2.6 Platelet aggregation assay

0.5 mL of human PRP obtained from Gulf Coat Blood Bank was incubated with 30 µL cells for 2 minutes. 10 μL (3 μmol/L) of AA was added to PRP solution to induce platelet aggregative. The aggregative activities were monitored by aggregometer (CHRONO‐LOG, PA) at a real‐time mode. The readings obtained indicated the percentage of platelet aggregation induced by each of the treated samples. For the assay using diluted platelets, PRP was diluted using platelet‐poor plasma (PPP), which was the supernatant of PRP after centrifugation for 20 minutes at 2400 *g*.

### 2.7 Transgenic mice

The transgenic mice were generated as previously described.^13^, ^26^

### 2.8 Tail‐cut arterial bleeding assay

A 0.5 cm tip of mouse tail was cut with a scissor. The arterial bleeding was blotted on a filter paper with a 10 seconds interval. The total bleeding time was calculated as the following formula:

Bleeding time (s)=number of dots×10.

## 3 RESULTS

### 3.1 Molecular modelling for TXAS and Co‐ordination between upstream COX‐1 and downstream TXAS, PGIS and mPGES‐1 in the bleeding site

**Figure 1:**
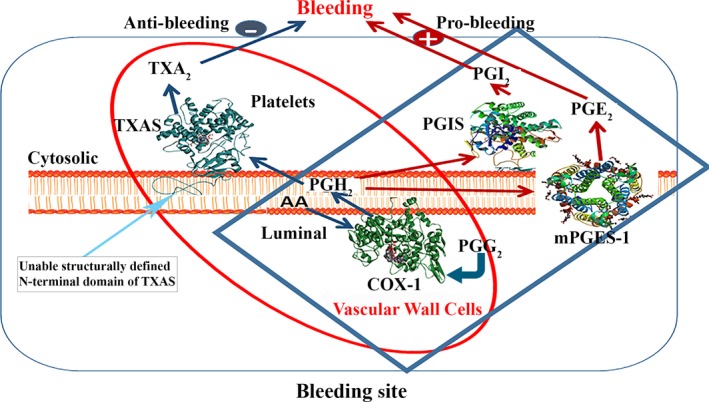
Schematic presentation of COX‐1 co‐ordinated with downstream three enzymes to maintain the balance of haemostasis. 3D crystal structures of human COX‐1 (PDB ID: 3N8Z^28^), PGIS (PDB ID: 3B6H^25^) and mPGES‐1 (PDB ID: 5T37^29^) used were downloaded from PDB. 3D structural model of human TXAS was created by homology modelling using the crystal structure of PGIS as a template. The co‐ordination of the upstream COX‐1 and downstream TXAS, PGIS and mPGES‐1 on ER membrane was schematically presented. A model demonstrating the three‐step catalytic activities: AA converted to PGG_2_, then PGH_2_ and finally to biologically active anti‐bleeding mediator TXA_2_, and the bleeding mediators PGE_2_ and PGI_2_ were shown

**Figure 2:**
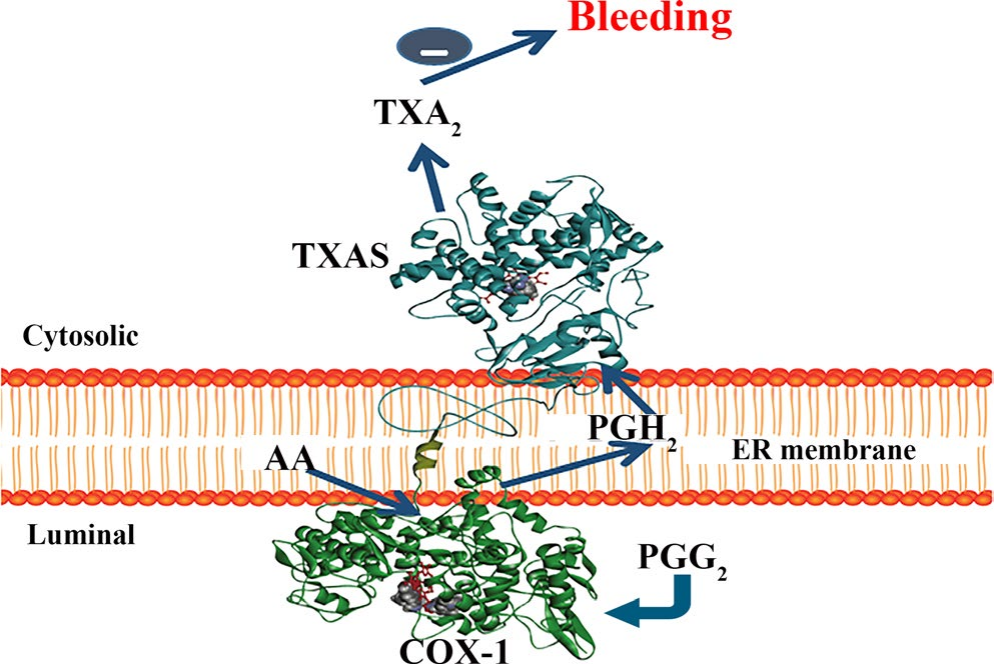
Design of a novel SCHEC. COX‐1‐10aa‐TXAS was created by linking the C‐terminus of COX‐1 to N‐terminus of TXAS through a linker, which contains 10‐amino acid residues (−10aa). The complete structural model of the COX‐1‐10aa‐TXAS with triple catalytically biological activities to convert AA to PGG_2_, then PGH_2_ and a final anti‐bleeding TXA_2_ within a single polypeptide chain were demonstrated

To perform structure‐based enzyme complex engineering, analysing 3D structures of the enzymes is a key step. So far, the 3D crystal structures for COX‐1,^18^, ^27^, ^28^ PGIS^29^ and mPGES^30^ have been solved. However, the crystal structure of TXAS is not available yet. In this study, we created a 3D structure model for human TXAS through the homology modelling approach, using the crystal structure of human PGIS as a template, which has highest similarity and identity with human TXAS (Figures 1 and 2). It shall also be indicated that because of the lack of crystal structure for the N‐terminal membrane domain of PGIS, the structural model of the N‐terminal domain of TXAS was unable to be defined (using a curved line as a schematic presentation, Figures 1 and 2). Crystallographic studies of detergent‐solubilised COXs suggested that the catalytic domain of the protein lies on the luminal side of the endoplasmic reticulum (ER, Figure 1) and is anchored to the ER membrane by the hydrophobic side chains of the amphipathic helices A‐D. These hydrophobic domains also form an entrance channel for the substrate AA^25^, ^27^, ^28^ (Figure 1). On the other hand, the results of our topological and structural studies performed through immunostaining and homology modelling led to the suggestion that the TXAS was anchored to the cytoplasmic side of the ER towards COXs^29^, ^30^, ^31^, ^32^ (Figure 1). It is also known that the C‐terminal domain of COX‐1 is towards the N‐terminal domain of TXAS on the ER. After bleeding occurs, the PGH_2_ produced by COX‐1 from AA is isomerized into the anti‐bleeding prostanoid TXA_2_ by TXAS, on ER cytosolic side in platelets and vascular cells (Figure 1, red circle). However, in these vascular cells, the PGH_2_ could also be isomerized into the bleeding contributors, PGI_2_ and PGE_2_, by PGIS and PGES in the ER environment, respectively (Figure 1, blue rectangle), thus increasing the production of TXA_2_, while decreasing the production of PGI_2_ and PGE_2_ should be able to increase the anti‐bleeding effect (Figure 1). This has led to the consideration of engineering an enzyme complex to control onsite AA metabolism in favour of TXA_2_, while disfavouring PGI_2_ and PGE_2_.

### 3.2 Design of a SCHEC, COX‐1‐10aa‐TXAS, performing three‐step reactions continually to control cellular AA metabolism into TXA_2_ specifically

In considering that the substrate channels of COX‐1 and TXAS open on opposite sides of the ER, it is safe to propose that the two channels held a very short distance from each other. In respect to the spatial structure and distribution of the substrate channels of COX‐1 and TXAS, a single‐chain hybrid enzyme complex (SCHEC), COX‐1‐10aa‐TXAS, which contained the two enzyme domains, was created as shown in Figure 2. COX‐1‐10aa‐TXAS holds an extension of the C‐terminal domain of COX‐1, through a transmembrane helical sequence, being the 10‐residue amino acid linker (His‐Ala‐Ile‐Met‐Gly‐Val‐Ala‐Phe‐Thr‐Trp) and then anchoring to the N‐terminus of TXAS. This design was aimed to shorten the travelling pathway of PGH_2_, from COX‐1 to TXAS, compared with that being passed to other free PGIS and PGES.

### 3.3 Creating a novel cDNA of COX‐1‐10aa‐TXAS using PCR approach and subcloning of the cDNA into an expression vector

**Figure 3:**
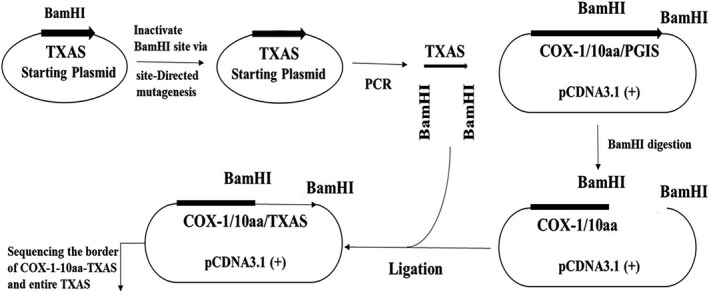
Construction and subcloning of the cDNA of COX‐1‐10aa‐TXAS into a mammalian expression vector, pcDNA3.1(+). The starting plasmid of TXAS and pcDNA3.1(+)‐COX‐1‐10aa‐PGIS vector was prepared previously.^13^ Full cDNA sequence of human TXAS was prepared by PCR after inactivated the BamHI site of the TXAS plasmid via site‐directed mutagenesis. On the other hand, the PGIS cDNA fragment was removed from the cDNA of COX‐1‐10aa‐PGIS, which was inserted in a pcDNA 3.1(+) vector. Next, the isolated TXAS cDNA was linked to the cDNA of the COX‐1‐10aa and then ligated to form a complete expression plasmid containing COX‐1‐10aa‐TXAS cDNA

A cDNA encoded the protein sequence of the SCHEC, COX‐1‐10aa‐TXAS, was prepared by PCR and subcloning approaches. First, the full cDNA of human TXAS was obtained by PCR using the previously created pcDNA3.1(+)‐TXAS as a template (Figure 3). On the other hand, the cDNA of human PGIS was removed and kept in the cDNA of COX‐1‐10aa, intact from the previously created pCDNA3.1(+)‐COX‐1‐10aa‐PGIS vector (Figure 3). Finally, the cDNA of TXAS was ligated with the cDNA of COX‐1‐10aa on the pCDNA3.1(+) vector to create a new expression vector (Figure 3), which is suitable for expression of human COX‐1‐10aa‐TXAS in mammalian cells. The detailed steps including the vectors, cutting sites, PCR and subcloning were illustrated in Figure 3.

### 3.4 Identification of stable expression of the recombinant COX‐1‐10aa‐TXAS in mammalian cells using Western blot

**Figure 4:**
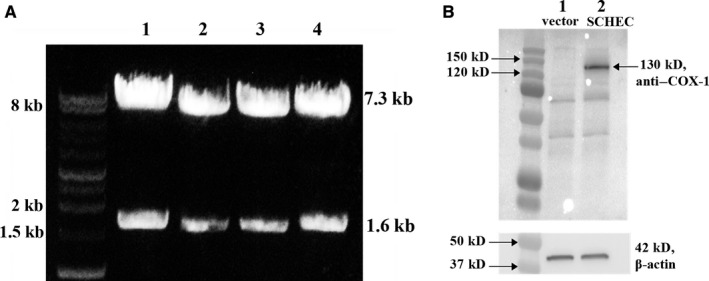
A, Verification of the cDNA of COX‐1‐10‐TXS inserted in pCDNA3.1(+) vector. Restriction enzyme digestion was performed to verify the successful insert of the sequence of cDNA of COX‐1‐10aa‐TXAS in the pcDNA3.1(+) vector. The restriction enzyme BamHI was used and cut twice at the two recognition sites and got two DNA fragments: one was the pcDNA3.1(+) vector with COX‐1‐10aa, and the other one is the TXAS. The sizes of the vector plus COX‐1‐10aa should be equal to 7.3 kb (5.4 kb of vector +1.9 kb of COX‐1‐10aa), and the size of TXAS cDNA should be equal to 1.6 kb. Finally, the subcloned cDNA sequencing of COX‐1‐10aa‐TXAS in the plasmid was further verified by DNA sequencing the inserted region of 10aa‐TXAS (data not showed). B, Western blot analysis to verify the expression of SCHEC in HEK293 cells. The HKE293 cells were transfected with the pcDNA3.1(+) vector contained cDNA of COX‐1‐10aa‐TXAS and then screened for stable expression using G418 up to 60 d. Approximately 20 µg of the proteins of HEK293 cells was subjected to Western blot analysis using 10% PAGE gel and anti–COX‐1 antibody. The results were shown on (B) panel: left, markers; lane 1, negative control, HEK293 cells transfected with the empty vector; lane 2, HEK293 cells transfected with the COX‐1‐10aa‐TXAS plasmid. The correct size of the expressed COX‐1‐10aa‐TXAS with approximately 130 kDa was indicated. β‐actin was used to normalize the protein concentration for the different samples

First, restriction enzyme digestion was used to cut out the cDNA fragment, which contained the encoded entire TXAS and the unique linker, 10aa, to verify the insert of the cDNA of COX‐1‐10aa‐TXAS within the cloned pcDNA3.1‐COX‐1‐10aa‐TXAS vector. The correct size (1.6 kb) of the insert for the fragment of COX‐1‐10aa‐TXAs cDNA and remaining size (7.3 kb) of the vector isolated from four clones were confirmed (Figure 4A). To test the expression of the recombinant COX‐1‐10aa‐TXAS in mammalian cells, the HEK293 cells were transfected with the cloned pcDNA3.1(+)‐COX‐1‐10aa‐TXAS vector. After 48 hours, the transfected HEK293 cells were further maintained and selected using G418 antibiotic to obtain the HEK293 cells stably expressing the COX‐1‐10aa‐TXAS. The expression of the SCHEC was firstly confirmed by Western blot analysis using anti–COX‐1 antibody (Figure 4B, lane 2). The HEK293 cells transfected with pcDNA3.1(+) vector were only used as control (Figure 4B, lane 1).

### 3.5 Further identification of the stable expression and subcellular localization of the COX‐1‐10aa‐TXAS in HEK293 cell line using immunocytochemistry approach

**Figure 5:**
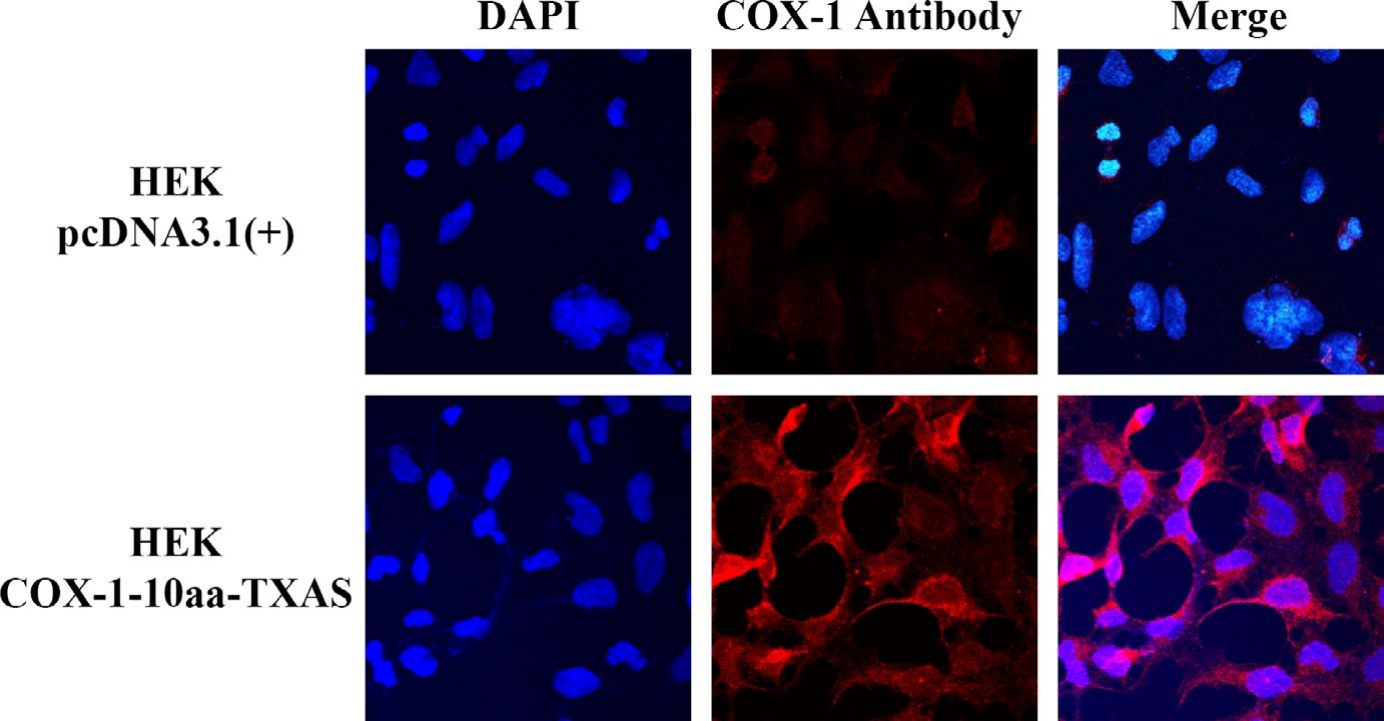
Immunofluorescent imaging of the expressed COX‐1‐10aa‐TXAs on HEK293 cells. The cells were cultured in the cover slides and transfected with cDNA: pcDNA3.1(+) empty vector (negative control [top]) or pcDNA3.1(+)‐COX‐1‐10aa‐TXAS (bottom). The cells were permeabilized by saponin and then incubated with mouse anti‐COX‐1 antibody. The bound primary antibodies were stained by rhodamine‐labelled goat antimouse IgG. The slides were imaged under a fluorescent microscope. Blue fluorescent DNA dye, DAPI, was used to image the nuclei of the cells

To further verify the stable expression of the recombinant COX‐1‐10aa‐TXAS in HEK293 cells, the stable cell line was subjected to be analysed using fluorescent immunocytochemistry. In addition, the high‐resolution immunostaining will be able to determine the subcellular localization of the expressed COX‐1‐10aa‐TXAS. The individual domain of COX‐1 (red) within the single polypeptide chain of COX‐1‐10aa‐TXAS was identified by immunostaining, using corresponding antibodies specifically bound to human COX‐1 (Figure 5, bottom panel, central) for the HKE293 cells expressing the SCHEC (Figure 5 bottom panel). In contrast, the HEK293 cells transfected with pcDNA3.1(+) vector only did not show any red endogenous COX‐1(Figure 5, top panel, central) staining. When merging the staining of the cell nuclei (DAPI, blue) and COX‐1 (red), the ER pattern staining for the expressed COX‐1‐10aa‐TXAS on the HEK293 cells were clearly identified (Figure 5, bottom panel). The data have strongly demonstrated that COX‐1‐10aa‐TXAS could be successfully expressed in mammalian cells and that the subcellular localization of the SCHEC is on the ER membrane, which is accordant with expectation, because the native COX‐1 and TXAS are localized on the ER membrane. The results suggested that the engineered COX‐1‐10aa‐TXAs had similar protein folding, post‐translational modification and ER topological arrangement as that of the wild‐type COX‐1 and TXAS in the mammalian cells. This also indicated that the expressed COX‐1‐10aa‐TXAS protein could be as stable as the wild types of individual enzymes with biological activities.

### 3.6 Establishing a reliable triple‐catalytic enzyme assay for the COX‐1‐10aa‐TXAS by using [^14^C]‐labelled arachidonic acid ([^14^C]‐AA) as the initial substrate

**Figure 6:**
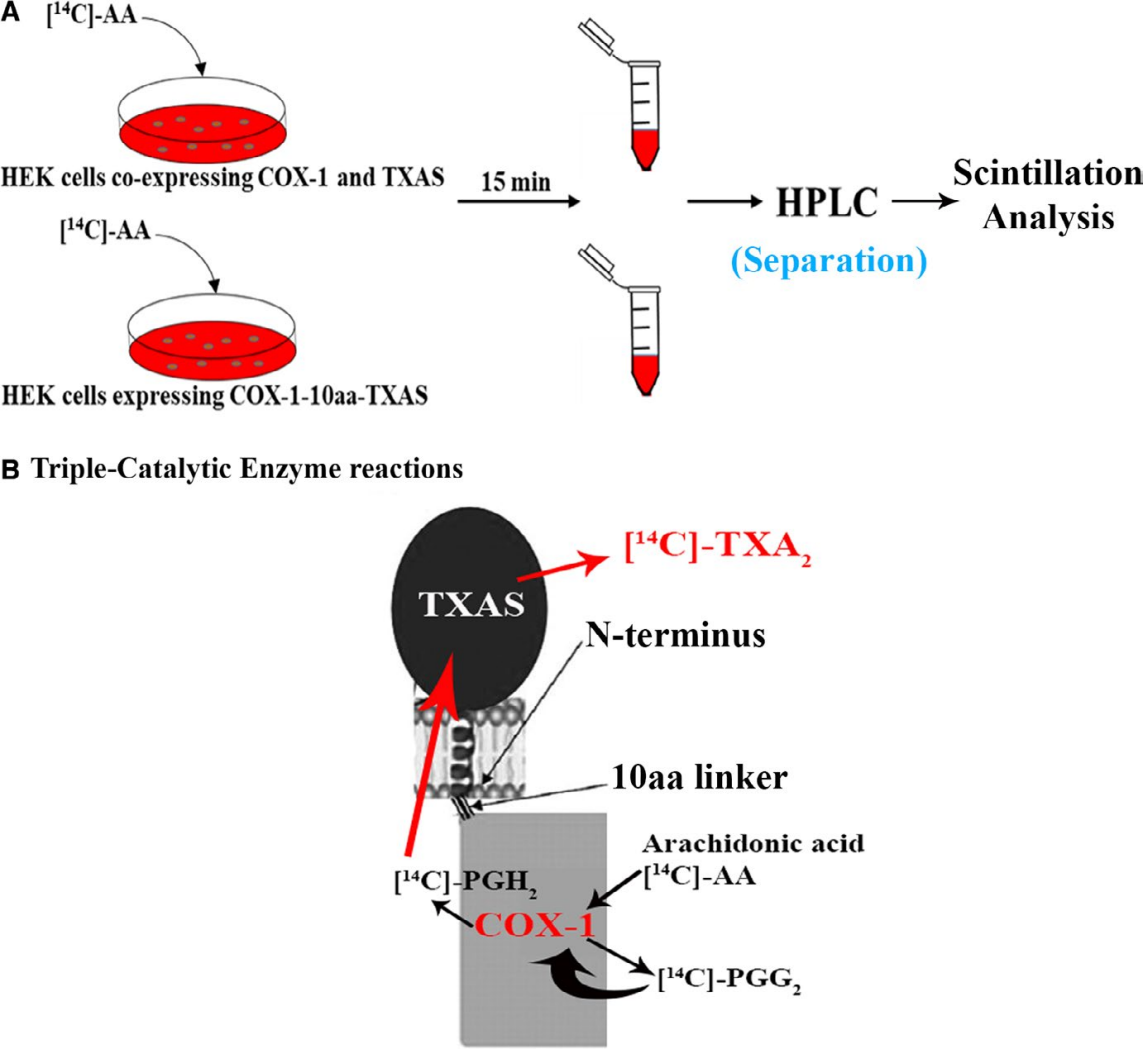
Schematic presentation for determination of the triple‐catalytic activities of the expressed COX‐1‐10aa‐TXAS using the highly sensitive and specific isotope‐labelled exogenous AA as a substrate. A, 3 µmol/L of [^14^C]‐AA was added to the HEK293 cells. After incubation for 15 min, the metabolized compounds from the [^14^C]‐AA were extracted out by buffer A (35% acetonitrile in 0.1% acetic acid) and then subjected to HPLC analysis using C‐18 column (4.5 × 250 mm) started with buffer A and then with a linear gradient of buffer B (100% acetonitrile) for 45 min. The eluents were directly connected to a scintillation analyzer to pick up the [^14^C]‐labelled compounds catalysed by the COX‐1‐10aa‐TXAS. B, [^14^C]‐mediators and [^14^C]‐products by the triple‐catalytic enzyme reactions of COX‐1‐10aa‐TXAS

As we mentioned above, directly converting AA into anti‐bleeding TXA_2_ while avoiding the side metabolites, such as the bleeding contributors, PGI_2_ and PGE_2_ (Figure 1), was the main characteristic of the novel COX‐1‐10aa‐TXAS based on our expectation. We tried to verify our hypothesis in HEK293 cells stably expressing COX‐1‐10‐aa‐TXAS (Figure 6A), through a highly sensitive and reliable assay. Under accurate monitoring by HPLC‐scintillation analyzer, this assay is able to profile [^14^C]‐AA to be metabolized into [^14^C]‐TXA_2_, through triple‐catalytic enzyme reactions (Figure 6B). The detailed assay conditions, steps and the reaction mechanisms were addressed in methods.

**Figure 7:**
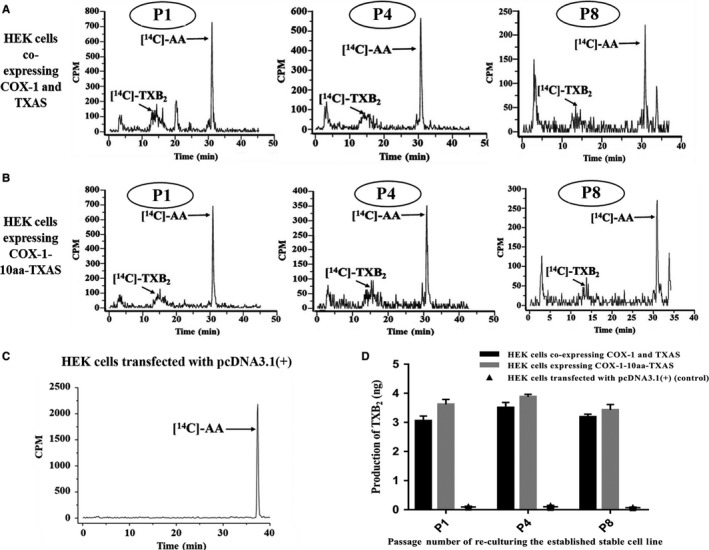
Determination of the activities of the HEK293 cells stably expressing COX‐1‐10aa‐TXAS and comparison with that of co‐expressing individual COX‐1 and TXAS. HEK293 cells were transfected with COX‐1‐10aa‐TXAS cDNA (B), or cotransfected with the individual COX‐1 cDNA and TXAS cDNA (A). After G418 screening for 2 mo, the cells were stored in the liquid nitrogen. After re‐cultured the cells, a serial of tests of the enzymatic activities of the cells were designed for P1, P4 and P8 cells. The cells firstly reaching 100% confluency were marked as P1 cells; 1 week later, after three passages, the cells with 100% confluency were marked as P4 cells; and another week for the P8 cells. The procedures described in Figure 6 were used to determine the [^14^C]‐products enzymatically metabolized from the added [^14^C]‐AA by the HEK293 cells expressing COX‐1‐10aa‐TXAS (B), or co‐expressed individual COX‐1 and TXAS (B). The peaks of [^14^C]‐TXB_2_ and its derivatives between 10 and 20 min of the retention time, representing the biologically active TXA_2_, were indicated with arrows labelled with [^14^C]‐TXB_2_. Metabolized [^14^C]‐AA with retention time (after 30 min) was also indicated in each assay. HEK293 cells transfected with pcDNA3.1(+) vector were used as a control (C). The quantitative analysis of the production of [^14^C]‐TXB_2_ from [^14^C]‐AA was shown in (D)

HEK293 cells stably expressing COX‐1‐10aa‐TXAS, and co‐expressing individual COX‐1 and TXAS (for positive control), were prepared by cDNA transfection and G418 screening up to 2 months and then stored in the liquid nitrogen. After re‐cultured the cells, serial tests of the enzymatic activities were accomplished for P1, P4 and P8 cells (Figure 7). The cells firstly reaching 100% confluency were marked as P1 cells; 1 week later, after three passages, the cells with 100% confluency were marked as P4 cells; and another week for the P8 cells. The triple‐catalytic activities of the HEK293 cells expressing COX‐1‐10aa‐TXAS (Figure 7B) were compared with that of HEK293 cells co‐expressing individual COX‐1 and TXAS, by the assay mentioned above. It should be indicated that [^14^C]‐TXA_2_ is not stable and can be further isomerized into a group of stable compounds, including [^14^C]‐TXB_2_ and its derivatives, which resulted in a group of broad peaks with retention time between 10 and 20 minutes (Figure 7A,B). However, determination of the generation of [^14^C]‐TXB_2_ and its derivatives using the HPLC‐scintillation method is the most reliable approach because it could exactly monitor the metabolism of the added [^14^C]‐AA distinguished from the endogenous AA. The data from Figure 7 have demonstrated that the HEK239 cells stably expressing COX‐1‐10aa‐TXAS could directly convert the [^14^C]‐AA to the final product [^14^C]‐TXB_2_ and its derivatives (Figure 7B), through the three steps of enzymatic reactions (Figure 6B), which were identical to that of the positive control (Figure 7A) that HEK293 cells co‐expressing individual COX‐1 and TXAS (Figure 7D). In contrast, the HEK293 cells transfected with pcDNA3.1(+) vector could not metabolize the [^14^C]‐AA at all (Figure 7C), which excluded the possibility of any endogenous COX‐1 and TXAS in the HEK293 cells. These results have confirmed that the expressed SCHEC, COX‐1‐10aa‐TXAS, has enzymatic activities to continuously convert AA into PGG_2_, then PGH_2_ and finally, TXA_2_.

### 3.7 Identification of the biological activities of COX‐1‐10aa‐TXAS to promote platelet aggregation in normal platelets and to rescue the aggregative functions of NSAIDs‐treated platelets

**Figure 8:**
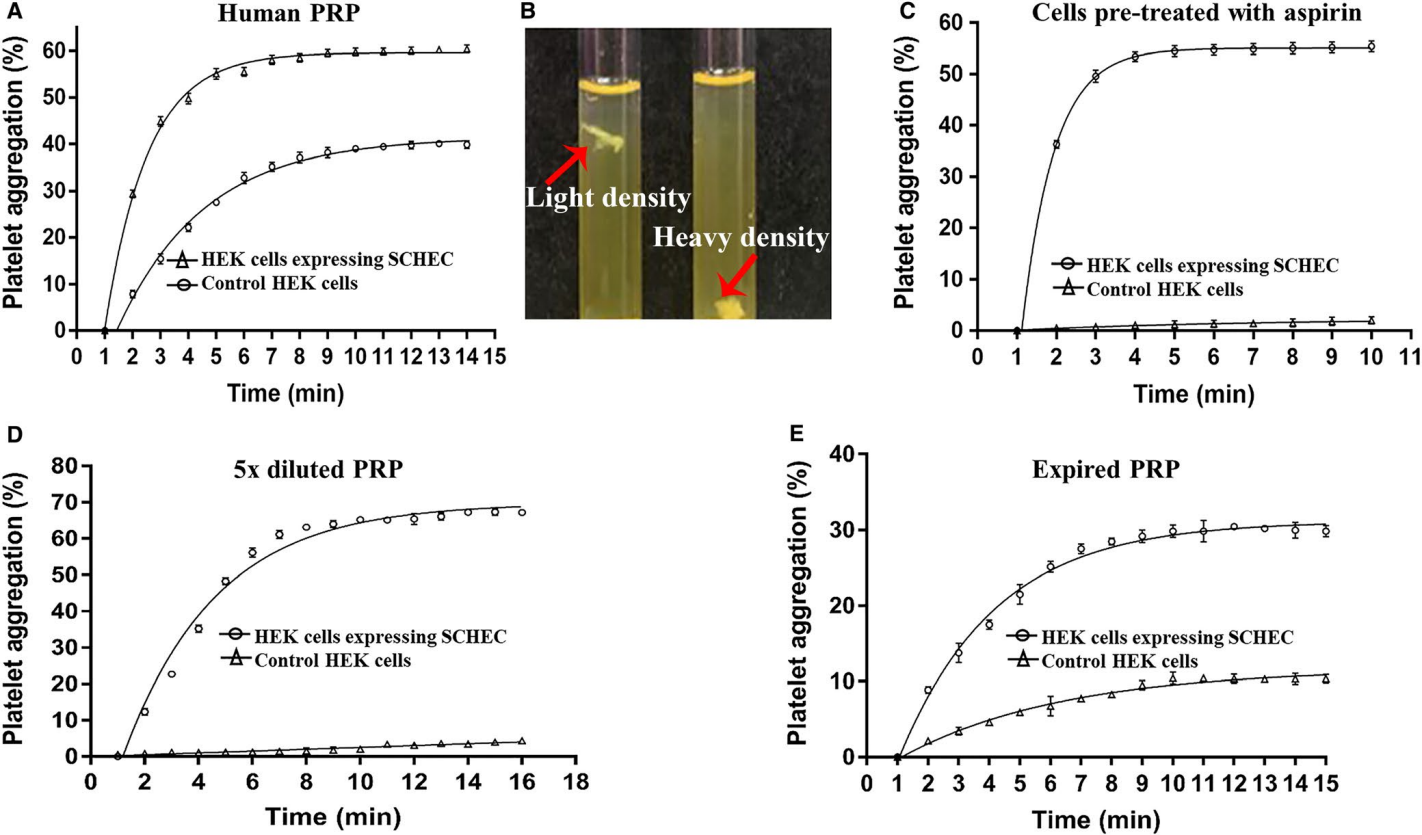
Determination of the biological activities of TXA_2_ produced by COX‐1‐10aa‐TXAS on promoting platelet aggregation. Human platelet‐rich plasma (PRP) obtained from the blood bank was used for the platelet aggregation tests. A, HEK293 cells expressing SCHEC (open triangles) or the HEK293 cells transfected with vector (HEK control, open circles) were incubated with PRP for 2 min. The aggregative activity was monitored by the addition of 10 µL AA (3 μmol/L of final concentration) using platelet aggregometer. The fibres (aggregated platelets) formed in the presence (B, right) or absence of SCHEC (B, left) were shown in (B). C, PRP was pre‐treated with 10 mmol/L aspirin for 20 min. The excessive aspirin was removed by centrifugation. The platelets were re‐suspended in clean platelet‐poor plasma (PPP) and then incubated with HEK293 cells expressing COX‐1‐10aa‐TXAS (open circles) or HEK control cells (open triangles) for 2 min. The platelet aggregation was monitored by addition of AA as described above. D, PRP was diluted by PPP (ration 1:4, 5 X) to mimic the platelet‐deficient conditions. The platelet aggregation was monitored in the presence (open circles) or absence (open triangles) of the COX‐1‐10aa‐TXAS by addition of AA as described above. E, PRP was stored at 4°C for 45 d exceeded the expiration date. The expired PRP lost majority of platelet aggregation activity was incubated with the HEK293 cells with (open circles) or without (open triangles) expressing COX‐1‐10aa‐TXAS and then subjected to restoration of the platelet aggregation assay by addition of AA as described above

First, the COX‐1‐10aa‐TXAS dramatically promoting platelet aggregation was observed in AA‐induced platelet aggregation assay using normal platelet‐rich plasma (PRP, Figure 8A). In comparison with that of the HEK cells control, the maximal aggregation rate was increased from 38% to 60%, and the ½ time for the maximal aggregation was reduced from 3.2 to 1.8 minutes by the HEK cells expressing COX‐1‐10aa‐TXAS (Figure 8A). The results are also supported by the observation that the aggregated platelets, in the presence of COX‐1‐10aa‐TXAS, exhibited a much heavier and solid form (Figure 8B, right) than that of the control (Figure 8B, left).

Administration of NSAIDs, such as aspirin, is one of the major causes of emergency bleeding situations. To test how the engineered SCHEC can rescue the NSAIDs‐resulted platelet dysfunction, the platelets were treated with aspirin, and then, the excessive aspirin was removed by washing with PBS. The recovery of platelet aggregative functions was compared between the absence and presence of the SCHEC in HEK cells. As a result, the aspirin‐treated platelets lost almost 100% of aggregative function in the absence of COX‐1‐10aa‐TXAS (Figure 8C, triangles), but they restored almost full aggregative function in the presence of the HEK293 cells expressing COX‐1‐10aa‐TXAS (Figure 8C, circles).

### 3.8 Further characterizing the biological activities of COX‐1‐10aa‐TXAS by using the approach of facilitating the platelet aggregation in the mimicked platelet‐deficient bleeding

In many clinical situations, the deficiency of platelets caused by diseases, such as chemotherapies, could be a complication with the possibility of life‐threatening bleeding. To mimic the platelet deficiency, we diluted the PRP to generate a concentration of the platelets being only 20% of the normal level. We applied the diluted PRP to the HEK293 cells expressing the SCHEC, as well as the normal HEK293 cells used as a control. The results demonstrated that COX‐1‐10aa‐TXAS could effectively promote the platelet aggregation under the deficient platelet concentration (Figure 8D). The results suggested that the COX‐1‐10aa‐TXAS has great potential to be developed into a novel type of bio‐enzymatic treatment for the platelet‐deficient bleeding.

### 3.9 Restoring aggregative functions of the expired PRP by COX‐1‐10aa‐TXAS

In general, the aggregative activities of platelets can only last for a couple of weeks under 4°C storage. To test the potential application to extend and/or restore the aggregative functions of platelets by using the SCHEC, the human PRP were tested after 45 days at 4°C storage. The responses of the expired platelets to AA‐stimulated platelet aggregation were very weak (Figure 8E, triangles). However, in the presence of the SCHEC, the platelet aggregation functions were significantly restored (Figure 8E, circles). This suggested that the SCHEC might be used to extend the reasonable expiration days for functional PRP.

### 3.10 Identification of anti‐bleeding effect of the COX‐1‐10aa‐TXAS in vivo

**Figure 9:**
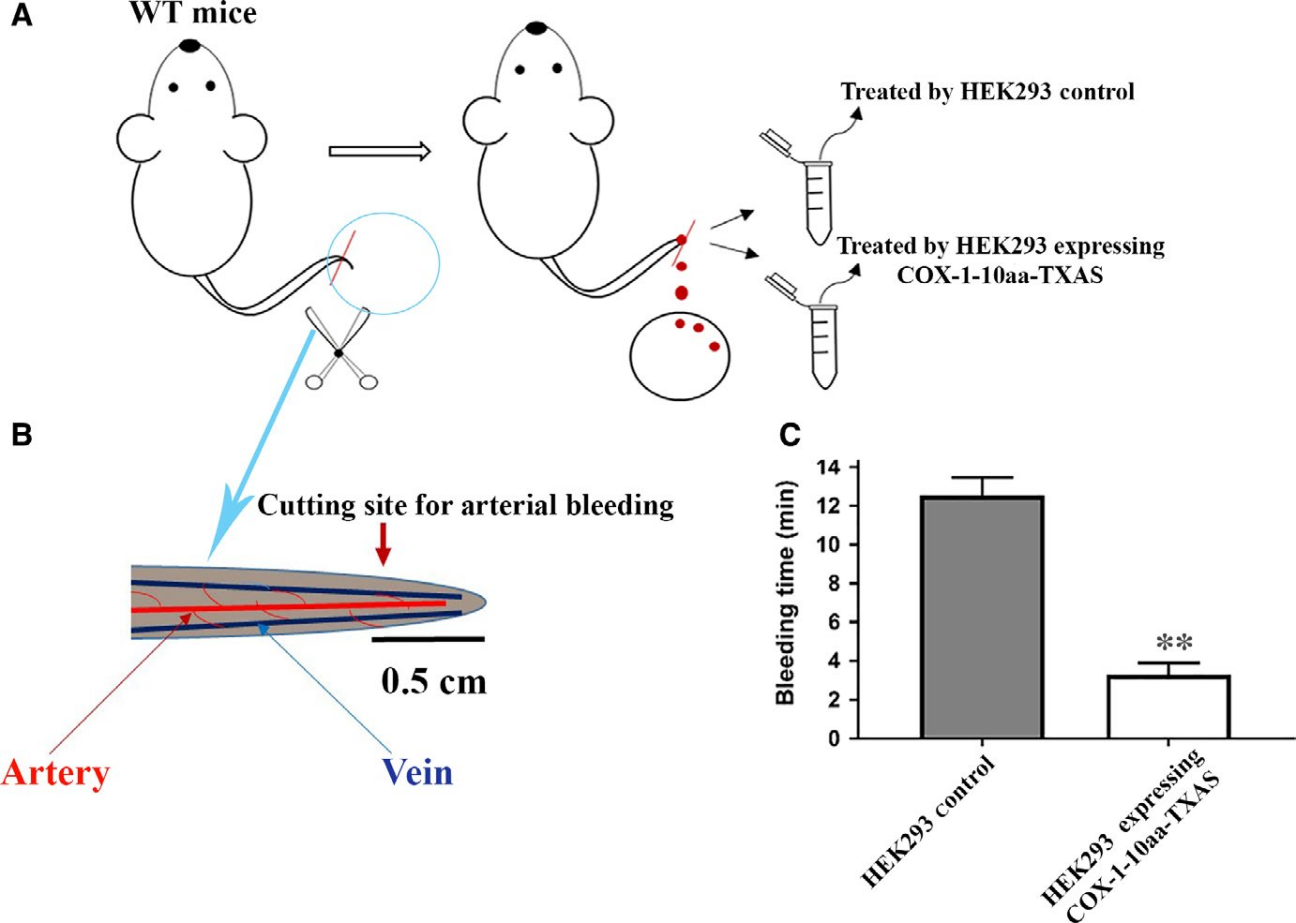
Examination of the anti‐bleeding effect of the expressed COX‐1‐10aa‐TXAs on bleeding site using tail‐cut arterial bleeding assay for normal mice in vivo. A, A 0.5 cm tip of mouse tail was cut by scissors to create an arterial bleeding site. The artery and vein displayed on the cutting site were shown in (B). Immediately, the bleeding tail was placed into the solution containing isolated microsomes of the HEK293 cells with (C, right) or without (C, left) expressing COX‐1‐10aa‐TXAS. The blood was blotted on a clean filter paper every 10 s until no detectable bleeding (A). The average and standard deviation of the bleeding time for each group (n = 6) were plotted in (C)

The tail‐cut bleeding assay was used to test the anti‐bleeding activity of the COX‐1‐10aa‐TXAS in vivo. The mice were divided into two groups: one group for the treatment, using HEK293 cells stably expressing COX‐1‐10aa‐TXAS, and another group for the control, using HEK293 cells transfected with vector only (HEK control, Figure 9A). An arterial bleeding site was created by cutting the tail arteries (0.5 cm from the end of tail) of the mice (Figure 9B) and then treated by applying the extracted microsomes of the HEK293 cells. The average arterial bleeding time was approximately 12 minute in the control group (Figure 9C, left). However, in the group treated by HEK293 cells expressing COX‐1‐10aa‐TXAS, the bleeding time was dramatically reduced to an average of 3.2 minutes (Figure 9C, right), which was highly significant. This result has clearly demonstrated that the engineered SCHEC had strong effects on anti‐arterial bleeding in vivo.

### 3.11 Further identification of the anti‐arterial bleeding of SCHEC in vivo by using a transgenic mouse model with a bleeding tendency

**Figure 10:**
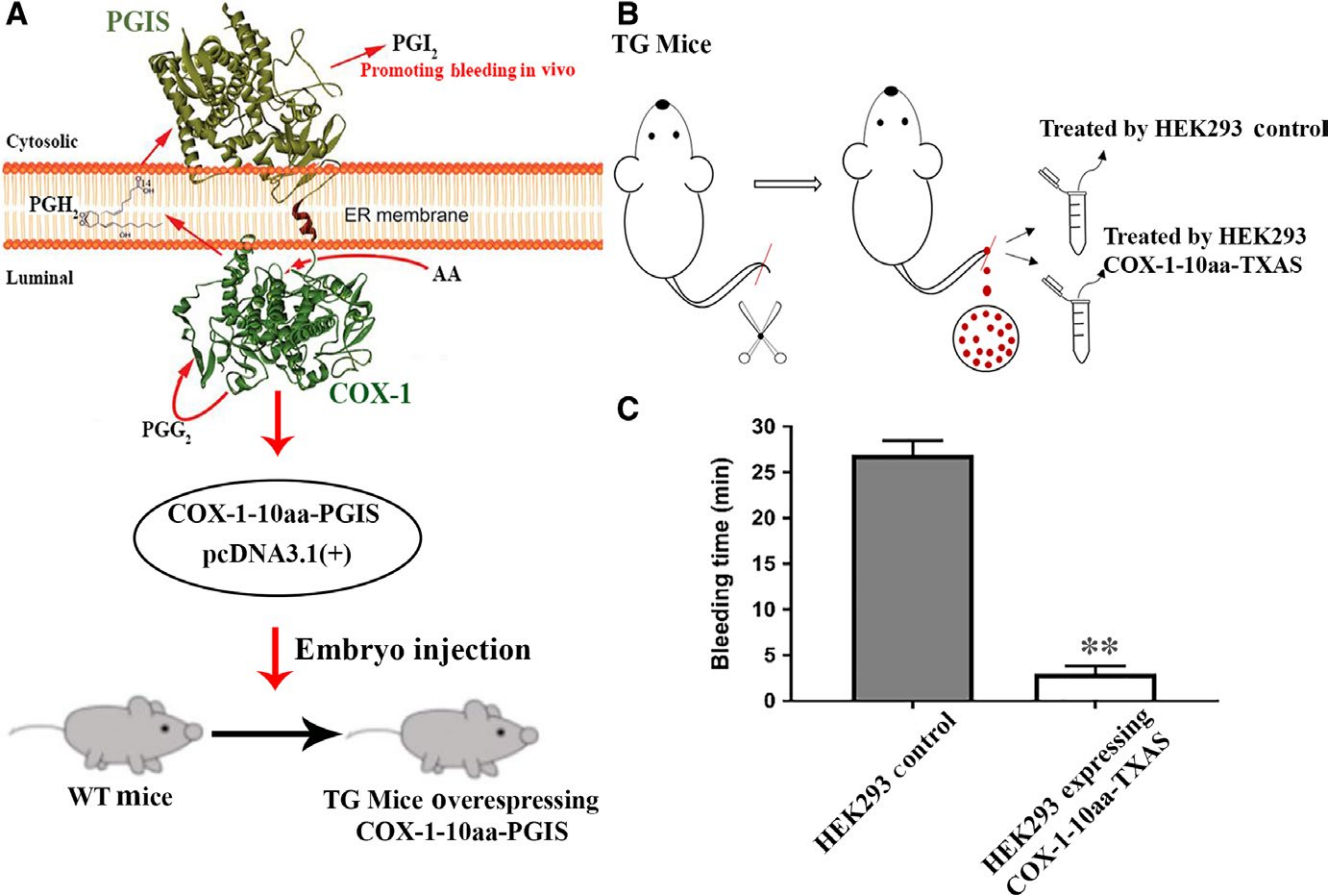
Examination of the anti‐bleeding effect of the expressed COX‐1‐10aa‐TXAs on bleeding site using tail‐cut arterial bleeding assay for transgenic mice with the bleeding tendency in vivo. A, The transgenic mice with bleeding tendency were created by injection of the cDNA of COX‐1‐10aa‐PGIS into the embryos of the mice (13). The arterial bleeding conditions were created by tail‐cut approach and then treated by the isolated microsomes of the HEK293 cells with (B, open bar) and without (B, closed bar) expressing COX‐1‐10aa‐TXAS. The averages and standard deviations of the bleeding times for each group (n = 6) were plotted (B)

Recently, we have successfully created a transgenic mouse model by overexpressing another engineered SCHEC designed by our group, COX‐1‐10aa‐PGIS in vivo.^13^ Among these transgenic mice, the overexpressed COX‐1‐10aa‐PGIS could directly convert AA into PGI_2_, which is a bleeding contributor with the effects of antiplatelet aggregation and vasodilation. The transgenic mice were created to prove that the COX‐1‐10aa‐PGIS is an effective enzyme complex to be against thrombotic stroke and ischaemia in vivo. The detailed procedures for creating and characterization of the transgenic mice were described previously.^13^ Briefly, a single‐chain cDNA of the COX‐1‐10aa‐PGIS was created and injected into the embryo to generate the transgenic mice (Figure 10A). Based on our previous studies, the transgenic mice demonstrated extended bleeding time as a result of effectively converting endogenous AA to PGI_2_, which inhibits platelet aggregation and promotes vasodilation; furthermore, the production of TXA_2_ was dramatically reduced in circulation in these transgenic mice. The arterial bleeding time was extended around twofold in the transgenic mouse model (an average of 28 minutes, Figure 10C, left), compared with that of the wild‐type mice (an average of 12 minutes, Figure 9C, left). After applying the microsomes of HEK293 cells expressing COX‐1‐10aa‐TXAS, the bleeding time of the transgenic mice was reduced to around 3 minutes (Figure 10C, right). These results demonstrated that the recombinant COX‐1‐10aa‐TXAS has the ability to overcome the TXA_2_ deficiency and rebalance the ratio of the TXA_2_ to PGI_2_ to stop bleeding in vivo.

## 4 DISCUSSION

The engineered SCHEC, COX‐1‐10aa‐TXAS, is capable of mimicing the triple‐catalytic activities of wild‐type COX‐1 and TXAS in converting the cellular AA to TXA_2_. Thus, this enzyme complex has provided very valuable information to understand the active ER configuration of the wild‐type COX‐1 and TXAS in the biosynthesis of TXA_2_. It has allowed us to predict that the physical distance between the native COX‐1 and TXAS on ER membrane is very similarly close to that of an enzyme complex, which has not been proposed yet. Crystallization of COX‐1‐10aa‐TXAS will be helpful to uncover the detailed structural and functional relationship between COX‐1 and TXAS in the PGH_2_ movement during the biosynthesis of the key molecule, TXA_2_, which is directly involved in the control of haemostasis as a blood clotting mediator.

There are several medical applications for the SCHEC, COX‐1‐10aa‐TXAS. First, it could be used to treat bleeding emergencies. One of the major advantages is that the engineered SCHEC is able to use endogenous cellular AA as a substrate. As a result of three‐step enzymatic reactions that occur continually and instantly to convert the released AA into TXA_2_ in the bleeding site, the SCHEC should be able to stop the bleeding on site effectively. Thus, the COX‐10aa‐TXAS has great potential to be developed into a biological reagent to treat bleeding in various bleeding emergencies. Second, the biological functions of TXA_2_ in mediating platelet aggregation and vasoconstriction in haemostasis are well characterized. However, its roles on other cells, such as neuronal and cancer cells, are poorly understood. Recent studies have reported that TXA_2_ demonstrates effects on promoting cancer cell proliferation,^21^, ^22^ and is also involved in post‐stroke–related neuronal cell damages.^33^ The success of engineering the active SCHEC, which specifically directed COX‐1–produced PGH_2_ to be passed to TXAS, was the first to make it possible to control cellular AA metabolism in favour to TXA_2_ and disfavouring other prostanoids. Transfection of the cDNA of COX‐1‐10aa‐TXAS to different cells, such as neuronal and cancer cells, could be used as models to uncover the roles of the TXA_2_ biosynthesis on the related diseases, such as neurodegeneration, and cancer development and metastasis.

Finally, overproduction of TXA_2_ by COX‐1 co‐ordinating TXAS is one of the major causes of thrombosis and vasoconstriction in ischaemic diseases, such as stroke, heart arrest, pulmonary arterial hypertension and deep vein thrombosis. Specifically, suppressing the production of TXA_2_ is an important step to rescue TXA_2_‐mediated ischaemia. One of the most common ways to prevent blood clotting is to apply low dose aspirin to reduce the production of TXA_2_. However, aspirin is a non‐selective COX inhibitor, which could also reduce the production of other important prostanoids, such as PGI_2_, which is a very important vascular protector that prevents damages from excess TXA_2_. It becomes more and more clear that aspirin may also promote cardiovascular diseases by inhibiting the generation of PGI_2_. However, the drug specifically inhibiting TXA_2_ production and maintaining normal PGI_2_ level is not available yet. With the combination of our previously developed COX‐1‐10aa‐PGIS with this newly engineered COX‐1‐10aa‐TXAS, we could use these complexes as targets for the screening of specific drugs, which only inhibit TXAS without affecting PGIS and COX‐1. Thus, this newly created COX‐1‐10aa‐TXAS could have a great impact if being used as a drug target for discovering the next generation of NSAIDs, which have fewer side effects on cardiovascular diseases.

In conclusion, the engineered SCHEC, COX‐1‐10aa‐TXAS, demonstrated the integrated triple‐catalytic reactions within just a single polypeptide, which could effectively convert endogenous AA into TXA_2_. These properties make it possible to control cellular AA metabolism to be in favour of TXA_2_ and disfavour of other prostanoids, such as PGI_2_ and PGE_2_. This controlled AA metabolism activity has exhibited effective anti‐bleeding properties. Furthermore, the SCHEC could be used as a specific target for screening anti‐stroke drugs, as well as a cellular model for understanding the relationship between TXA_2_ and other diseases, such as cancer and neuronal degeneration diseases. This SCHEC could be further used for understanding the topology, structure and functional relationships between the two enzymes, COX‐1 and TXAS, anchored to the ER membrane, during the biosynthesis of TXA_2_ (proposed in Figure 1).

## CONFLICT OF INTEREST

The authors declare no competing interests.

## AUTHOR CONTRIBUTIONS

Q‐YL, YL, Q‐LL, MH and S‐PS performed the experiments, analysed the data and prepared the figures. K‐HR designed the experiments, and YL prepared figures. YL and K‐HR wrote the manuscript. All authors reviewed the manuscript.

## References

[1] Hamberg M , Svensson J , Samuelsson B . Thromboxanes: a new group of biologically active compounds derived from prostaglandin endoperoxides. Proc Natl Acad Sci USA. 1975;72:2994‐2998. 1059088 10.1073/pnas.72.8.2994 PMC432905

[2] Ding X , Murray PA . Cellular mechanisms of thromboxane A2‐mediated contraction in pulmonary veins. Am J Physiol‐Lung Cell Mol Physiol. 2005;289:L825‐L833. 15964897 10.1152/ajplung.00177.2005

[3] Yamamoto K , Ebina S , Nakanishi H , Nakahata N . Thromboxane A2 receptor‐mediated signal transduction in rabbit aortic smooth muscle cells. Gen Pharmacol. 1995;26:1489‐1498. 8690235 10.1016/0306-3623(95)00025-9

[4] Smyth EM . Thromboxane and the thromboxane receptor in cardiovascular disease. Clin Lipidol. 2010;5:209‐219. 20543887 10.2217/CLP.10.11 PMC2882156

[5] Winn R , Harlan J , Nadir B , Harker L , Hildebrandt J . Thromboxane A2 mediates lung vasoconstriction but not permeability after endotoxin. J Clin Invest. 1983;72:911‐918. 6886010 10.1172/JCI111062 PMC1129256

[6] Narumiya S , Sugimoto Y , Ushikubi F . Prostanoid receptors: structures, properties, and functions. Physiol Rev. 1999;79:1193‐1226. 10508233 10.1152/physrev.1999.79.4.1193

[7] Vane JR . Inhibition of prostaglandin biosynthesis as the mechanism of action of aspirin‐like drugs. Adv Biosci. 2014;9:395‐411.

[8] Li C , Hirsh J , Xie C , Johnston M , Eikelboom J . Reversal of the anti‐platelet effects of aspirin and clopidogrel. J Thromb Haemost. 2012;10:521‐528. 22268852 10.1111/j.1538-7836.2012.04641.x

[9] Ricciotti E , FitzGerald GA . Prostaglandins and Inflammation. Arterioscler Thromb Vasc Biol. 2011;31:986‐1000. 21508345 10.1161/ATVBAHA.110.207449 PMC3081099

[10] Ruan K‐H , Mohite A , So S‐P , Ruan C‐H . Establishing novel prostacyclin‐synthesizing cells with therapeutic potential against heart diseases. Int J Cardiol. 2013;163:163‐169. 21722977 10.1016/j.ijcard.2011.06.007

[11] Deng Y , Yang Z , Terry T , et al. Prostacyclin‐producing human mesenchymal cells target H19 lncRNA to augment endogenous progenitor function in hindlimb ischaemia. Nat Commun. 2016;7:11276. 27080438 10.1038/ncomms11276 PMC4835554

[12] Zhou L , Chen Z , Vanderslice P , et al. Endothelial‐like progenitor cells engineered to produce prostacyclin rescue monocrotaline‐induced pulmonary arterial hypertension and provide right ventricle benefits. Circulation. 2013;128:982‐994. 23841984 10.1161/CIRCULATIONAHA.113.003139

[13] Ling Q‐L , Mohite AJ , Murdoch E , et al. Creating a mouse model resistant to induced ischemic stroke and cardiovascular damage. Sci Rep. 2018;8:1653. 29374184 10.1038/s41598-018-19661-y PMC5786049

[14] Ruan K‐H , Cervantes V , So S‐P . Engineering of a novel hybrid enzyme: an anti‐inflammatory drug target with triple catalytic activities directly converting arachidonic acid into the inflammatory prostaglandin E2. Protein Eng Des Sel. 2009;22:733‐740. 19850676 10.1093/protein/gzp058 PMC2777022

[15] Akasaka H , Ruan K‐H . Identification of the two‐phase mechanism of arachidonic acid regulating inflammatory prostaglandin E2 biosynthesis by targeting COX‐2 and mPGES‐1. Arch Biochem Biophys. 2016;603:29‐37. 27177970 10.1016/j.abb.2016.04.011

[16] Chillar A , So SP , Tang N , Ruan KH . Identification of tumorigenesis from the specific coupling of cyclooxygenase‐2 with microsomal prostaglandin E2 synthase‐1 in vivo. Eur J Inflamm. 2014;12:77‐88.

[17] Ruan D , So S‐P . Prostaglandin E2 produced by inducible COX‐2 and mPGES‐1 promoting cancer cell proliferation in vitro and in vivo. Life Sci. 2014;116:43‐50. 25139833 10.1016/j.lfs.2014.07.042

[18] Akasaka H , So S‐P , Ruan K‐H . Relationship of the topological distances and activities between mPGES‐1 and COX‐2 versus COX‐1: implications of the different post‐translational endoplasmic reticulum organizations of COX‐1 and COX‐2. Biochemistry. 2015;54:3707‐3715. 25988363 10.1021/acs.biochem.5b00339

[19] Egan KM , Lawson JA , Fries S , et al. COX‐2‐derived prostacyclin confers atheroprotection on female mice. Science. 2004;306:1954‐1957. 15550624 10.1126/science.1103333

[20] Castellana M , Wilson MZ , Xu Y , et al. Enzyme clustering accelerates processing of intermediates through metabolic channeling. Nat Biotechnol. 2014;32:1011. 25262299 10.1038/nbt.3018 PMC4666537

[21] Ruan KH , So SP , Cervantes V , Wu H , Wijaya C , Jentzen RR . An active triple‐catalytic hybrid enzyme engineered by linking cyclo‐oxygenase isoform‐1 to prostacyclin synthase that can constantly biosynthesize prostacyclin, the vascular protector. FEBS J. 2008;275:5820‐5829. 19021758 10.1111/j.1742-4658.2008.06703.x PMC3038792

[22] Huang D , Ren L , Qiu C‐S , et al. Characterization of a mouse model of headache. Pain. 2016;157:1744. 27058678 10.1097/j.pain.0000000000000578 PMC4960827

[23] Deng H , Wu J , So S‐P , Ruan K‐H . Identification of the residues in the helix F/G loop important to catalytic function of membrane‐bound prostacyclin synthase. Biochemistry. 2003;42:5609‐5617. 12741817 10.1021/bi026749z

[24] Ruan K‐H , Deng H , Wu J , So S‐P . The N‐terminal membrane anchor domain of the membrane‐bound prostacyclin synthase involved in the substrate presentation of the coupling reaction with cyclooxygenase. Arch Biochem Biophys. 2005;435:372‐381. 15708381 10.1016/j.abb.2004.12.018

[25] Ruan K‐H , Deng H , So S‐P . Engineering of a protein with cyclooxygenase and prostacyclin synthase activities that converts arachidonic acid to prostacyclin. Biochemistry. 2006;45:14003‐14011. 17115695 10.1021/bi0614277

[26] Ruan K‐H , Mohite AJ , So S‐P . Resistant to thrombosis, induced stroke and heart arrest by incorporation of a single gene of PGI2‐synthesizing COX‐1‐PGIS in vivo: Implication against human heart disease. Int J Cardiol. 2013;168:2960‐2961. 23602873 10.1016/j.ijcard.2013.03.114

[27] Luong C , Miller A , Barnett J , Chow J , Ramesha C , Browner MF . Flexibility of the NSAID binding site in the structure of human cyclooxygenase‐2. Nat Struct Mol Biol. 1996;3:927. 10.1038/nsb1196-927 8901870

[28] Picot D , Loll PJ , Garavito RM . The X‐ray crystal structure of the membrane protein prostaglandin H2 synthase‐1. Nature. 1994;367:243. 8121489 10.1038/367243a0

[29] Hui D , Huang A , Shui‐Ping S , Yue‐Zhen L , Ke‐He R . Substrate access channel topology in membrane‐bound prostacyclin synthase. Biochem J. 2002;362:545‐551. 11879180 10.1042/0264-6021:3620545 PMC1222417

[30] Ke‐He R , Shui‐Ping S , Zheng W , Jiaxin W , Dawei L , Jennifer K . Solution structure and topology of the N‐terminal membrane anchor domain of a microsomal cytochrome P450: prostaglandin I2 synthase. Biochem J. 2002;368:721‐728. 12193162 10.1042/BJ20021001 PMC1223024

[31] Wu J , So S‐P , Ruan K‐H . Determination of the membrane contact residues and solution structure of the helix F/G loop of prostaglandin I2 synthase. Arch Biochem Biophys. 2003;411:27‐35. 12590920 10.1016/s0003-9861(02)00728-2

[32] Ruan K‐H . Advance in understanding the biosynthesis of prostacyclin and thromboxane A2 in the endoplasmic reticulum membrane via the cyclooxygenase pathway. Mini Rev Med Chem. 2004;4:639‐647. 15279598 10.2174/1389557043403710

[33] Yu Q‐J , Tao H , Wang X , Li M‐C . Targeting brain microvascular endothelial cells: a therapeutic approach to neuroprotection against stroke. Neural Regen Res. 2015;10:1882‐1891. 26807131 10.4103/1673-5374.170324 PMC4705808

